# Risk and Protective Factors for PTSD in Caregivers of Adult Patients with Severe Medical Illnesses: A Systematic Review

**DOI:** 10.3390/ijerph17165888

**Published:** 2020-08-13

**Authors:** Claudia Carmassi, Claudia Foghi, Valerio Dell’Oste, Carlo Antonio Bertelloni, Andrea Fiorillo, Liliana Dell’Osso

**Affiliations:** 1 Department of Clinical and Experimental Medicine, University of Pisa, 56100 Pisa, Italy; claudia.carmassi@unipi.it (C.C.); foghi.claudia@gmail.com (C.F.); carlo.ab@hotmail.it (C.A.B.); liliana.dellosso@med.unipi.it (L.D.); 2 Department of Biotechnology, Chemistry and Pharmacy, University of Siena, 53100 Siena, Italy; 3 Department of Psychiatry, University of Campania “L. Vanvitelli”, 80100 Naples, Italy; andrea.fiorillo@unicampania.it

**Keywords:** Post-Traumatic Stress Disorder (PTSD), Post-Traumatic Stress Symptoms (PTSS), mental health burden, relatives, significant others, carers

## Abstract

Caregivers of severely ill individuals often struggle to adjust to new responsibilities and roles while experiencing negative psychological outcomes that include depression, anxiety and Post-Traumatic Stress Disorder (PTSD). This systematic review aims to outline potential risk and protective factors for the development of PTSD in caregivers of adult subjects affected by severe somatic, potentially life-threatening illnesses. Twenty-nine studies on caregivers of adult patients affected by severe, acute, or chronic somatic diseases have been included. Eligibility criteria included: full-text publications reporting primary, empirical data; PTSD in caregivers of adult subjects affected by severe physical illnesses; risk and/or protective factors related to PTSD; and English language. Specific sociodemographic and socioeconomic characteristics, besides the illness-related distress, familiar relationships, exposure characteristics, coping style, and support, were identified as relevant risk/protective factors for PTSD. The review limitations are the small number of studies; studies on different types of diseases; studies with same samples. It is crucial to consider factors affecting caregivers of severely ill adult patients in order to plan effective intervention strategies aimed at reducing the risk of an adverse mental health outcome and at enhancing the psychological endurance of this population.

## 1. Introduction

Caregiving burden is defined as the physical, psychological, social, or economic strain that caregivers may experience during the care of a loved one [1,2]. Caregivers are often overwhelmed in the early period of critical illness such that they struggle to understand even basic information about their loved one’s diagnosis, treatment, or prognosis [3]. Additionally, the role of family caregiver can be extremely stressful and result in many adverse outcomes, ranging from mild psychological stress to an increased risk of death [4]. Indeed, family caregivers, while struggling to adjust to new responsibilities and roles [5], may experience negative psychological outcomes that include new or worsening depression, anxiety, and Post-Traumatic Stress Disorder (PTSD) symptoms [6]. Furthermore, the prevalence of psychiatric disorders in caregivers can be associated to psychological symptoms in the patient [7,8,9], virtually worsening the patient outcome.

Increasing evidence on psychiatric consequences on caregivers of patients with mental disorders are available, some of which is on PTSD [10,11,12,13,14,15,16,17,18], however less data are available on caregiving burden of patients affected by severe somatic illnesses, mostly reporting on anxiety or depressive symptoms [19,20,21,22]. However, in a public health perspective it is extremely relevant to investigate PTSD in such population. PTSD, in fact, is usually related to reduced quality of life, increased risk of other psychopathological conditions [23,24,25,26,27], substance abuse [28], and considerable costs for healthcare systems [29]. These data were also confirmed by the Authors of the European Study of the Epidemiology of Mental Disorders Survey (ESEMeD), who reported that this kind of traumatic event largely contributed to the European 12 months PTSD prevalence [30].

Conversely, scant data are still available on PTSD in caregivers of patients affected by potentially life-threatening or severe acute or chronic somatic diseases and, even though some risk factors contributing to this disorder have been reported across studies [31], additional research is required to determine how robust is their association with PTSD. While it is important to identify risk factors, it is also very important to detect protective factors for PTSD symptoms. 

Therefore, the aim of this systematic review was to analyze the risk and protective factors for PTSD symptoms in caregivers of adult patients with life-threatening or severe medical somatic illnesses, in order to promote healthcare services’ awareness of PTSD at-risk subjects and to enhance possible protective or supportive factors.

## 2. Materials and Methods 

### 2.1. Literature Search

A systematic search was conducted from 1 May 2020 to 1 August 2020. Studies were identified by searching the electronic databases MEDLINE (Pubmed), EMBASE, and Ovid by using the combination of search terms (PTSD OR posttraumatic stress OR post-traumatic stress disorder OR post-traumatic stress symptoms OR PTSS) AND (caregiver OR caregivers OR carer OR carers) without filters, restrictions or limits. All studies from 1 January 1990 to 1 August 2020 were contemplated in the databases search. Furthermore, we checked the reference lists from the reviews emerging from our search, in order to find additional relevant studies. 

### 2.2. Eligibility Criteria

Studies adhering to the following criteria were included in this review:

1. Full-text publications reporting primary, empirical data explicitly relating to PTSD symptoms in caregivers of adult subjects affected by severe physical illnesses (i.e., somatic, chronic, acute, or life threatening diseases).
2. Articles that found possible risk and/or protective factors related to PTSD symptoms in caregivers.
3. Articles available in English.

### 2.3. Screening and Selection Process

C.F. (Claudia Foghi) and V.D.O. (Valerio Dell’Oste) conducted the studies selection process. The primary databases search yielded a total of 2943 records. Following the removal of 2879 publications after titles and abstracts screening because articles were duplicates *(n =* 1753), or irrelevant *(n* = 951), or resulted not available or not in English *(n* = 30), or were other article types *(n* = 145), 64 articles passed the initial screening. We retrieved and read the full text journal articles, and these articles were screened against eligibility criteria. Both a first (C.F.) and a second (V.D.O.) rater independently completed the process. Any discrepancy highlighted during the categorization process was discussed and consensus reached. The grade of agreement between the two authors was good. Any disagreements about inclusion or exclusion of studies were discussed and resolved by a third author (C.C. (Claudia Carmassi)).

**Figure 1:**
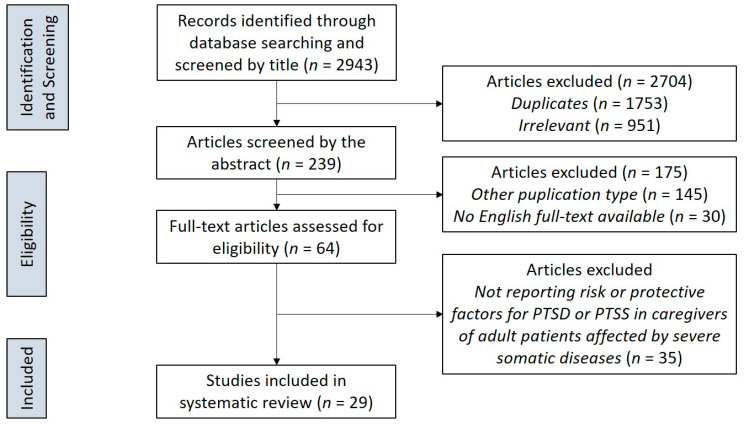
PRISMA flowchart of the study selection process. PRISMA, Preferred Reporting Items for Systematic reviews and Meta-Analyses; PTSD, Post-Traumatic Stress Disorder; PTSS, Post-Traumatic Stress Symptoms.

Thirty-five studies were excluded because of not highlighting significant risk or protective factors for PTSD symptoms in caregivers of adult patients affected by severe somatic diseases, so that a total of 29 studies were identified for inclusion in the review. Decisions for inclusion or exclusion are summarized in a flow chart according to Preferred Reporting Items for Systematic reviews and Meta-Analyses (PRISMA) recommendations (see Figure 1). PRISMA is an evidence-based manual for reporting in systematic reviews and meta-analyses and focuses on the reporting of reviews evaluating randomized trials, but it can also be used as a basis for reporting systematic reviews of other types of research [32].

### 2.4. Quality Assessment

The quality of the 29 included studies was assessed by means of the Quality Assessment Tool for Observational Cohort and CrossSectional Studies (QATOCCSS) [33]. Each study was scored as either “good,” “fair,” or “poor” (see Table 1). Two independent reviewers (C.F. and V.D.O.) performed the quality assessment and a third reviewer (C.A.B. (Carlo Antonio Bertelloni)) cross-checked quality assessment results. Disagreements were discussed and resolved with the research team.

## 3. Results

Upon our search, 29 studies were identified for inclusion in the review. Details of each study included in the review are provided in Table 1. Particularly, PTSD prevalence rates ranged from 4.17% [34] to 54% [35] and clinically significant PTSD symptoms rates ranged from 11% [36] to 74.1% [37].

### 3.1. Risk Factors for PTSD.

For what concerns the risk factors for PTSD in caregivers, studies highlighted the role of sociodemographic and socioeconomic characteristics; familiar relationships; illness-related distress; exposure characteristics; and psychiatric symptoms and negative/maladaptive coping.

*Sociodemographic and socioeconomic characteristics.* Females resulted to be more affected by PTSD symptoms in many studies [38,39,40,41,42,43,44,45,46]. Younger caregivers also seemed to suffer from higher PTSD symptoms in three studies [35,47,48]. Other studies reported lower income as risk factor for developing PTSD symptoms [38,49]. Another study on 151 partners of patients coping with an acute coronary syndrome found that a lower level of education was associated to higher PTSD symptoms [36], and similar results emerged from another study [39]. Only one study on 103 family caregivers of neurologic intensive care unit (neuroICU) patients reported an association between being not married nor cohabitating and higher PTSD symptoms [44]. Andresen et al. [40] in a sample of 83 close relatives of ICU patients, found that older patient’s age was associated to higher PTSD symptoms in relatives. This trend was shown up to patient’s age of 62 years old, and then there was no more increase. Conversely, Hartog et al. [41], examining 84 relatives of ICU patients, found that younger patient’s age was associated with higher PTSD symptoms. 

*Familiar relationships.* Having a closer relationship with patients (i.e., being spouse or parent) was a factor related to PTSD symptoms in a study on 163 family caregivers of adult patients with acute leukemia [50]. Another study on 31 spouses and 25 close relatives of hospitalized patients with acute burns found that spouses had significantly higher levels of PTSD symptoms than close relatives [44]. Similarly, Hartog et al. [41] found that spouses were more prone to have higher PTSD symptoms in comparison to children and other relatives. Some other studies have examined the role of family relationships. Two studies have shown that having a lower bond or a poor relationship with the patient increased the risk of developing PTSD symptoms in the caregivers [47,51]. Teixeira and Pereira [42], analyzing a sample of 214 adult children caregivers of cancer patients, found that having a more enmeshed and chaotic family functioning predicted higher PTSD symptoms.

*Illness-related distress*. Many disease-related factors have been found to enhance PTSD symptoms. The uncertainty related to disease and the family strain have been related to PTSD symptoms in a study on 333 caregivers of hematopoietic cell transplant recipients [52]. Richardson et al. [53] found that in 78 caregivers of patients with head and neck cancer, having perceptions of low benefits from treatment and the presence of many patient symptoms, increased the risk of experiencing symptoms of PTSD in caregivers. Another study on 214 adult children caregivers of cancer patients highlighted that the perception of higher patient dependency was associated to higher PTSD symptoms [54]. Similarly, caregiving burden and caregiving strain have been stressed as a potential risk factors for PTSD in other studies [47,55,56]. Rumpold et al. [55] in a prospective study on 80 family caregivers of advanced cancer patients, found that caregiver subjective burden at baseline was significantly associated with PTSD symptoms at 9 months follow-up. Another study on 36 caregivers of ICU patients found that caregiving strain, represented by emotional adjustment, social issues, and physical and financial strain, was associated to increased PTSD symptoms [56]. Some authors [40], investigating a sample of 83 close relatives of ICU patients, found a relationship between patient’s Acute Physiology and Chronic Health Evaluation (APACHE) II score, an ICU scoring system used to classify the severity of disease, and PTSD symptoms in caregivers. Indeed, when the patient’s APACHE II score rose from 7 to 20, there was an increase in PTSD symptoms in caregivers, even though afterward the trend flattened. Other studies found an association between a greater severity of the disease [39], more days of hospitalization [40], persistent patient’s pain [49], and the levels of PTSD symptoms. Another study on 82 family members of ICU patients found that being caregivers of ICU patients with a traumatic brain injury (TBI), rather than of ICU patients without TBI, was a risk factor for experiencing more PTSD symptoms [57]. Furthermore, Dew et al. [47] examined 190 family caregivers to heart transplant recipients and found that in the first year post-transplant, caregivers presented higher PTSD symptoms. A more recent study on informal caregivers of adult hematopoietic cell transplant recipients showed that a shorter time since transplant was associated with greater PTSD symptoms in caregivers [52]. Moreover, Teixeira and Pereira [54] found that a shorter disease and caregiving duration were associated with a poorer outcome in terms of PTSD symptomatology, while Carek et al. [48], examining 51 caregivers of recent stroke survivors, found that an increased time since the event, with consequently more chronic stressors related to the sequelae of the medical event, was related to higher PTSD symptoms. Finally, Norup and Elklit [39] found that also the subjective evaluation of severity of illness may have enhanced PTSD symptoms. 

*Exposure characteristics*. In a study on 41 family members of patients in the neuroICU, researchers found that having had more frequent visits in the aftermath of the event, which implied more time spent at bedside, was related to greater PTSD symptoms [49]. Having accompanied the patient during the drive to the Intensive Cardiac Care Unit (ICCU) after witnessing the medical event also was found to be a risk factor for PTSD, in a study on 143 female partners of acute coronary event patients [37]. Finally, in a prospective study on 102 relatives of patients with chronic obstructive pulmonary disease who survived an ICU stay, peritraumatic dissociation was related to higher PTSD symptoms at 90 days post-discharge [58].

*Psychiatric symptoms and negative/maladaptive coping*. Alfheim et al. [35], analyzing a sample of 211 family caregivers of ICU patients, found that having more comorbidities, such as depression or pain, was predictive of having more PTSD symptoms. Presenting higher levels of anxiety [51,59], depression [58], or both depression and anxiety symptoms [39,43] were all found to be related to a higher severity of PTSD symptoms. As concerns the prior psychiatric history, having a history of depression [43,45], depressive and anxiety disorders [47], or more generically a personal positive history for psychiatric illness [38] were associated with increased PTSD symptoms. Even the presence of psychiatric symptoms in patients was found to be a risk factor for some authors, particularly patient’s PTSD symptoms in three studies [50,52,60] and panic disorder symptoms in another study on 168 caregivers of advanced cancer patients [34]. Negative coping styles have been related to greater PTSD symptoms severity in a study on 86 family members and friends of patients who have suffered spontaneous subarachnoid hemorrhage [61]. Another two studies have highlighted the importance of maladaptive coping strategies, such as avoidance, denial, behavioral disengagement and use of humor, in predicting caregivers’ PTSD symptoms [47,53]. Finally, a study on 101 relatives of out-of-hospital cardiac arrest patients found that caregivers’ perception of patient’s therapy as insufficient was related to higher PTSD symptoms [45].

### 3.2. Protective Factors for PTSD. 

Social status, familiar relationships, support, and positive coping resulted the most important factors related to lower PTSD symptoms in caregivers of severely ill adult patients. 

*Social status*. Alfheim et al. [35] found that being on sick leave was a risk factor for PTSD and suggested that continuing to work reduced PTSD symptoms in caregivers. Also having higher educational levels was associated with fewer PTSD symptoms [40].

*Familiar relationships*. Being the parent of the patient, instead of the spouse or a friend [35] was found to be related to a better outcome in PTSD symptoms. Moreover, Stukas et al. [38], examining a sample of 142 family caregivers of heart transplant recipients, found that having a higher level of family cohesiveness reduced PTSD symptomatology.

*Support*. The importance of support has been highlighted by many studies [38,39,43,50,54,62]. Norup and Elklit [39], examining 614 partners of people with epilepsy, found that a high level of social support decreased PTSD symptoms, and similar findings were highlighted in another study on 39 partners of head and neck cancer survivors [43]. Another study on 306 surrogate decision makers of patients with chronic critical illness found that perceptions of clinician support and communication reduced PTSD symptoms [62].

*Positive coping*. A positive coping style [44] and mindfulness [44,51] were both shown to reduce PTSD symptoms. Indeed, Choi et al. [51], on a sample of 99 caregivers of patients admitted to a neuroICU, found that caregivers with higher levels of mindfulness were more likely to have lower PTSD symptoms. Having feelings of hope [35] or a perceived sense of mastery in the situation [47] were also associated with a reduction in PTSD symptoms.

## 4. Discussion

The present review summarizes relevant risk or protective factors for PTSD symptoms in caregivers of patients affected by severe or life-threatening somatic diseases that emerged across studies, besides the increasing evidence of high rates of this disorder and its symptoms in caregivers. 

In line with previous literature across different study populations, female caregivers were found to be more affected by PTSD symptoms [38,39,40,41,42,43,44,45,46] corroborating female gender as a major risk factor [63,64,65,66,67]. Other studies [35,47,48] found that older family caregivers reported fewer PTSD symptoms, suggesting the role of different coping strategies and life experiences with respect to younger ones. 

Negative/maladaptive coping styles were reported as risk factors for PTSD in three studies [47,53,61]. Richardson et al. [53] found denial and/or behavioral disengagement in caregivers were related to higher PTSD symptoms. Indeed, these are avoidant coping strategies characterized by behaviors that promote escape from stressful situations and the related negative emotions [68]. Avoidant coping behaviors show significant overlap with PTSD symptoms and prevent recovery from the disorder, as they are associated with increased symptom severity over time [69,70,71]. This is likely because avoidant coping strategies prevent actions aimed at actively managing stressors, potentially resulting in a paradoxical increase in intrusive thoughts [72,73]. 

For what concerns protective factors, some authors [35] found that being on sick leave increased PTSD symptoms in caregivers. Even though this might mean that those experiencing the highest levels of PTSD symptoms are unable to also manage work, as sustained by previous research [74,75], we may also argue that work was a protective factor. Thus, holding a job outside the home might potentially serve both as a caregiver further stressor and as a factor associated with improved mental health, maybe providing additional social and financial supports and giving the opportunity to respite from caregiving responsibilities. 

Another interesting finding was that having a higher level of family cohesiveness was protective against PTSD symptomatology [38], highlighting perceptions of friendliness and supportiveness between members of the family as protective. More widely, the importance of family and social support has been highlighted by many other studies [19,22,38,39,43,50,54,62]. Wendlandt et al. [62] also found that support and communication from healthcare personnel reduced PTSD symptoms in caregivers. For this, education resources should be made available to assist clinicians in enhancing their communications skills.

Furthermore, we found that using a positive coping style [44], having higher levels of mindfulness [44,51], and having feelings of hope [35] were all associated with a reduction in PTSD symptoms. Indeed, teaching mindfulness-based stress management, while reducing distress, could bolster psychological and behavioral resilience [76]. Moreover, hope has been reported to be the most prominent theme in helping family caregivers to believe in a positive outcome despite uncertainties with the situation [77], and methods to strengthen hope have been described in cancer patients [78], even though there need to be further research on caregivers of various medical contexts. 

Having a perceived sense of mastery in the situation [47] was also associated with a reduction in PTSD symptoms. Moreover, in accordance with previous studies [79], Fait et al. [36] reported that a lower level of education was associated to higher PTSD symptoms, and similar findings were reported by other two studies [39,40]. We may argue that they potentially correlate with ability to understand complexities that may be inherent in caring responsibilities, such as engage with health professionals and comprehending medical information.

The identification of risk factors for PTSD symptoms in caregivers of severely ill adult individuals could help in detecting subjects most likely to develop post-traumatic stress reactions. Together with the awareness of potential protective factors, this could allow healthcare services to plan effective intervention strategies and supportive measures aimed at mitigating the psychological impact of caregiving burden and at enhancing resilience. Indeed, assessment of risky factors could help in making an early intervention in more vulnerable subjects, with a possible improvement in PTSD outcome [80,81,82]. Furthermore, interventions aimed at enhancing support and positive coping strategies could nourish caregiver resilience, which is fundamental to face traumatic events [82,83,84,85]. 

When discussing our results some limitations had to be taken into account. The first one is the relatively small number of studies. Second, the inclusion of eight studies assessed as being of ‘poor’ quality, so that findings from these studies may have had a less significant correlation with PTSD symptomology. Third, we found studies on different types of severe diseases, with different prognosis, which could account for a difference in psychic burden on family caregivers. Finally, the inclusion of some studies with same samples, in some cases with different data analysis in the same study sample (i.e., [42] and [54]), and in other cases with one study sample extended with respect to the previous one (i.e., [38] and [47]). 

## 5. Conclusions

A caregiving role of severely ill adult patients can be extremely stressful and could result in an array of adverse outcomes, including PTSD. Identification of risk factors that are modifiable, such as support and communication, and the recognition of not modifiable risk factors, such as sex, may be important to allow providers to prospectively identify caregivers who are vulnerable to develop psychological disorders, as PTSD, and then to target them with focused interventions. Clinicians could indeed provide prompt effective support to caregivers by recognizing the magnitude of their perceived stress and by developing novel and effective supportive strategies. Developing guidelines for family-centered care and support in wards treating severely ill patients could help nourishing psychological wellbeing of caregivers and, possibly, of patients.

## Figures and Tables

**Table 1:** Characteristics of included studies.

Study	Year	Study Type	Sample	Quality Rating	Assessments	PTSD Rates	Risk Factors	Protective Factors
Alfheim et al. [35]	2018	Longitudinal	211 family caregivers of intensive care unit patients	Good	IES-r	PTSD: 54% (at enrolment); 24% (at 12 months)	-Younger -Having more comorbidities (e.g., pain, depression.) -Being on sick leave	-Being the parent of the patient (than spouse or friend) -Increased levels of hope -Working
Andresen et al. [40]	2015	Prospective	83 caregivers of ICU patients assessed at admission (time I), at 2 to 4 days (time II) and 60 days (time III).	Fair	PCLS	PTSD: 22.89%	-Older patient’s age, up to 62 years old -Greater severity of disease from APACHE II level of 7 up to 20 -More days of hospitalization -Female	-Educational level
Bambauer et al. [34]	2006	Longitudinal	168 patient–caregiver dyads (Advanced cancer patients and their primary, informal, non-paid caregivers)	Fair	SCID IV	PTSD: 4.17%	-Patient psychiatric disorders	
Bond et al. [46]	2017	Prospective	31 spouses and 25 close relatives of hospitalized patients with acute burns	Good	Modified PTSD Symptom Scale	PTSD: 23.21% at admission, 8.33% at discharge	-Women -Spouses	
Carek et al. [48]	2010	Cross-sectional	51 informal caregivers of recent stroke survivors	Poor	PDS; PTCI	PTSD: 20%	-Younger -Increased time since stroke	
Choi et al. [51]	2018	Prospective, observational	99 caregivers of patients admitted to neuroICU assessed during admission (baseline), three months, and six months post-hospitalization	Good	PCLS	PTSD: 16% (baseline); 22% (at six months)	-Fearful/anxious profile during admission -Negative relationship with patient	-Strong positive relationship with patient -Mindfulness
Cornelius et al. [37]	2020	Longitudinal	143 female partners of acute coronary event patients four months after the event	Fair	PDS-5	PTSD symptoms: 74.1%	-Accompanying the patients during the drive to the hospital, than only witnessing the emergence of symptoms.	
De Miranda et al. [58]	2011	Prospective multicenter	102 informal caregivers of patients with chronic obstructive pulmonary disease	Fair	IES	PTSD: 29.8% (on day 90)	-Peritraumatic dissociation at ICU discharge -Depressive symptoms	
Dew et al. [47]	2004	Prospective	190 caregivers to heart transplant recipients evaluated at 2, 7, 12, and 36 months post-transplant	Good	CIDI	PTSD-T: 22.5% (cumulative rates 3 years post-transplant)	-Younger -Lower bond with patient -Caregiver burden -First year post-transplant -History of depressive and anxiety disorders -Maladaptive coping	-Higher sense of mastery
Fait et al. [36]	2016	Cross-sectional	151 partners of patients with acute coronary syndrome 2 to 6 months after patients’ hospitalization	Poor	PC-PTSD	CDI-PTSD symptoms: 11%	-Lower level of education	
Hartog et al. [41]	2015	Prospective observational	84 relatives od ICU patients interviewed by phone after 90 days after patients had died or were discharged	Fair	IES	PTSD: 51%	-Younger patient age -Female -Spouses (with respect to children and other relatives)	
Jia et al. [50]	2015	Cross-sectional	163 caregivers of adult patients with acute leukemia	Poor	PCL-C	PTSD symptoms: 36.8%	-Closer relationship with patients (e.g., spouses) -Higher levels of patients’ PTSS	-Higher levels of perceived social support
Liang et al. [52]	2019	Cross-sectional	333 caregivers of adult hematopoietic cell transplant recipients	Poor	PCL-5	PTSD: 6.6%	-Shorter time since hematopoietic cell transplant. -PTSD in patient -Higher cancer-related distress	
McPeake et al. [56]	2016	Cross-sectional	36 caregivers of ICU patients	Poor	IES	PTSD:53%	-Caregiving strain	
Meyers et al. [44]	2020	Longitudinal prospective cohort	103 family caregivers of neuroICU patients at baseline and 3- and 6-month follow-up	Good	PCL-Specific Stressors	PTSD symptoms: 16% baseline; 14% at 6 months	-Female -Not married/cohabitating	-Higher baseline mindfulness -Positive coping
Moschopoulou et al. [43]	2018	Cross-sectional	39 partners of head and neck cancer survivors	Fair	PCL-C	PTSD: 12.8%; PTSD symptoms: 25.7%	-Prior history of depression -Female -Symptoms of depression and anxiety	-Social support
Noble and Schenk [61]	2008	Cross-sectional	86 family members and friends of patients with spontaneous subarachnoid hemorrhage	Fair	PDS	PTSD: 25.6%	-Maladaptive coping strategies	
Norup and Elklit [39]	2013	Cross-sectional	614 partners of people with epilepsy	Poor	HTQ	full PTSD: 7.7%; partial PTSD: 43.9%	-Female -Less years of education -Objective and subjective epilepsy severity -Anxiety and depression	-Social support
Richardson et al. [53]	2016	Prospective	78 caregivers of patients with head and neck cancer at diagnosis and 48 caregivers six months later	Good	PSSSR	PTSD: 19%	-Perceived little benefit from treatment -Many cancer symptoms -Denial and/or behavioral disengagement at diagnosis	-Use of humor at diagnosis
Rumpold et al. [55]	2016	Prospective	80 family caregivers of advanced cancer patients at baseline and at 9 months follow-up	Good	IES-r	PTSD: 19.5% (baseline); 12.5% (follow-up)	-Caregiver subjective burden	
Stukas et al. [38]	1999	Prospective	142 family caregivers of heart transplant recipients	Good	CIDI	PTSD: 7.7%	-Female -Younger -Lower income -Personal history of psychiatric disorder -Lower friend support	-Higher family cohesiveness
Sundararajan et al. [59]	2014	Cross-sectional	63 family members of ICU patients	Fair	IES-r	PTSD symptoms: 41.2%	-Anxiety symptoms	
Teixeira and Pereira [54]	2012	Cross-sectional	214 adult children caregivers of parents with cancer	Poor	IES-r	Not reported	-Female -Perception of higher parental dependency -Shorter disease and caregiving’s duration	-Social support
Teixeira and Pereira [42]	2016	Cross-sectional, comparative	214 adult children caregivers of cancer patients and 78 adult children of nonchronically ill parents	Poor	IES-r	Not reported	-Female -Enmeshed or chaotic family functioning	
Trevick and Lord [49]	2017	Prospective cohort	41 caregivers of neuroICU patients at baseline, 26 at 1 month and 23 at 6 months	Fair	IES-r	PTSD: 7.7% at 1 month and 17% at 6 months	-Lower income -More frequent visits at 1 month -Persistent pain at 6 months	
Warren et al. [57]	2016	Longitudinal	40 family members of ICU patients with traumatic brain injury (TBI) and 42 of non-TBI ICU patients, assessed at baseline and 3 months	Good	PC-PTSD	PTSD symptoms: 24.3%	-ICU patients with TBI vs. ICU patients without TBI	
Wendlandt et al. [62]	2019	Randomized controlled	306 surrogate decision makers of patients with chronic critical illness 90 days post-randomization	Good	IES-r	Not reported		-Support and communication
Wintermann et al. [60]	2019	Cross-sectional	70 partners of chronically critically ill patients	Fair	PTSS-10	PTSD symptoms:18.6%	-Patient’s PTSS	
Zimmerli et al. [45]	2014	Observational	101 relatives of out-of-hospital cardiac arrest patients	Good	IES-r	PTSD: 40%	-Females -History of depression -Family perception of patient’s therapy as insufficient	

APACHE II, Acute Physiology And Chronic Health Evaluation II; CIDI, Composite International Diagnostic Instrument; HTQ, Harvard Trauma Questionnaire; ICU, Intensive Care Unit; IES, Impact of Event Scale; IES-r, Impact of Event Scale-Revised; PCL-5, PTSD Checklist for the Diagnostic and Statistical Manual of Mental Disorders 5th edition; PCL-C, PTSD Checklist-Civilian Version; PCLS, Post-traumatic stress disorder checklist, version S; PC-PTSD, Primary care-PTSD screening questionnaire; PDS, Post-traumatic Diagnostic Scale; PDS-5, Post-traumatic Diagnostic Scale for DSM-5; PSSSR, Post-Traumatic Stress Disorder Symptom Scale; PTCI, Post-traumatic Cognitions Inventory; PTSD, Post-Traumatic Stress Disorder; PTSD-T, Post-Traumatic Stress Disorder related to the Transplant; PTSS, Post-Traumatic Stress Symptoms; PTSS-10, Post-traumatic Stress Scale; SCID-IV, Structured Clinical Interview for DSM-IV.
